# Dynamic life-cycle carbon analysis for fast pyrolysis biofuel produced from pine residues: implications of carbon temporal effects

**DOI:** 10.1186/s13068-021-02027-4

**Published:** 2021-09-29

**Authors:** Kai Lan, Longwen Ou, Sunkyu Park, Stephen S. Kelley, Prakash Nepal, Hoyoung Kwon, Hao Cai, Yuan Yao

**Affiliations:** 1 Department of Forest Biomaterials, North Carolina State University, 2820 Faucette Drive, Raleigh, NC 27606 USA; 2 Systems Assessment Center, Energy Systems Division, Argonne National Laboratory, 9700 South Cass Avenue, Lemont, IL 60439 USA; 3 USDA Forest Service, Forest Products Laboratory, 1 Gifford Pinchot Drive, Madison, WI 53726 USA; 4 Center for Industrial Ecology, Yale School of the Environment, Yale University, 380 Edwards Street, New Haven, CT 06511 USA

**Keywords:** Life-cycle carbon analysis, Dynamic modeling, Carbon accounting, Biofuel, Fast pyrolysis, Pine residues, Monte Carlo simulation

## Abstract

**Background:**

Woody biomass has been considered as a promising feedstock for biofuel production via thermochemical conversion technologies such as fast pyrolysis. Extensive Life Cycle Assessment studies have been completed to evaluate the carbon intensity of woody biomass-derived biofuels via fast pyrolysis. However, most studies assumed that woody biomass such as forest residues is a carbon–neutral feedstock like annual crops, despite a distinctive timeframe it takes to grow woody biomass. Besides, few studies have investigated the impacts of forest dynamics and the temporal effects of carbon on the overall carbon intensity of woody-derived biofuels. This study addressed such gaps by developing a life-cycle carbon analysis framework integrating dynamic modeling for forest and biorefinery systems with a time-based discounted Global Warming Potential (GWP) method developed in this work. The framework analyzed dynamic carbon and energy flows of a supply chain for biofuel production from pine residues via fast pyrolysis.

**Results:**

The mean carbon intensity of biofuel given by Monte Carlo simulation across three pine growth cases ranges from 40.8–41.2 g CO_2_e MJ^−1^ (static method) to 51.0–65.2 g CO_2_e MJ^−1^ (using the time-based discounted GWP method) when combusting biochar for energy recovery. If biochar is utilized as soil amendment, the carbon intensity reduces to 19.0–19.7 g CO_2_e MJ^−1^ (static method) and 29.6–43.4 g CO_2_e MJ^−1^ in the time-based method. Forest growth and yields (controlled by forest management strategies) show more significant impacts on biofuel carbon intensity when the temporal effect of carbon is taken into consideration. Variation in forest operations and management (e.g., energy consumption of thinning and harvesting), on the other hand, has little impact on the biofuel carbon intensity.

**Conclusions:**

The carbon temporal effect, particularly the time lag of carbon sequestration during pine growth, has direct impacts on the carbon intensity of biofuels produced from pine residues from a stand-level pine growth and management point of view. The carbon implications are also significantly impacted by the assumptions of biochar end-of-life cases and forest management strategies.

**Supplementary Information:**

The online version contains supplementary material available at 10.1186/s13068-021-02027-4.

## Background

Cellulosic biofuel produced from renewable biomass has great potential to enhance energy security while reducing the environmental impacts of the transportation sector [1–3], which accounts for 29% of the total US emissions (6457 million metric ton CO_2_e) in 2017 [4]. The US Renewable Fuel Standard mandates that 44% of the US total renewable fuel derived from cellulosic biofuels in 2022, and that these cellulosic biofuels achieve at least a 60% reduction in life-cycle greenhouse gas (GHG) emissions compared to the 2005 petroleum baseline [5]. The potential energy and environmental benefits of cellulosic biofuel, which is critical for decision-making related to biofuel policy, research, development, and commercialization, are often quantified through Life Cycle Assessment (LCA) covering cradle-to-grave stages for biofuel production associated with various feedstocks and conversion technologies [6–26]. Among many conversion technologies, fast pyrolysis has attracted the most attention for its ability to produce a suite of fuel products from a wide variety of biomass feedstocks. Fast pyrolysis is a thermochemical conversion technology that rapidly decomposes organic compounds under temperature between 400–600 ℃ in an oxygen-limited atmosphere to create a crude bio-oil that can be further refined into fuel products [27]. Most LCA models have implemented fast pyrolysis processes based on different lignocellulosic feedstocks, including corn stover, switchgrass, miscanthus, and pine residues [15, 16, 28–33].

Forest residues generated in thinning, logging, and wood product manufacturing are one of the most abundant feedstocks in the US. According to the Billion-Ton Study by the US Department of Energy (US DOE) [34], there are potentially 30–108 million oven dry metric tons of forest residues available each year. This resource is currently underutilized as it is either left on-site or much less commonly burned for energy recovery [34, 35]. There has been a growing interest in converting forest residues to biofuels to enhance the efficient utilization of forest resources, reduce the risks of forest wildfire, and bring additional revenue to landowners [36–39]. A few studies have conducted LCA for forest residue-derived biofuel and indicated a significant reduction (36–67%) of life-cycle GHG emissions compared to conventional fuels [10, 14, 15, 32, 33]. Nonetheless, most studies have not assessed the impacts of feedstock variations that are particularly important for woody biomass given the wide diversity of forest types, growing regions, forest management practices, and harvesting alternatives. More importantly, few LCA studies have investigated carbon temporal effects or examined the carbon neutrality assumption for biofuel derived from woody biomass that is generally considered carbon–neutral despite its much longer growth cycle compared to annual crops or perennial biomass. Addressing all these issues is critical to developing sustainable strategies for forest management as the carbon emitted from the decay and the combustion of forest residues, and carbon stored in durable wood products are temporally dynamic and highly driven by forest management strategies that may also influence biofuel production. To quantify the carbon temporal effects, several studies have proposed corresponding Global Warming Potential (GWP) accounting methods and showed the necessity of considering carbon dynamics related to bioenergy products [40–47]. For example, in 2011, Cherubini et al. [40] proposed a method to estimate the GWP of CO_2_ emissions from biomass combustion based on impulse response functions (IRF) of CO_2_, and showed that the current static assumption or carbon neutrality assumption should be revised by considering the carbon dynamics in bioenergy system. Levasseur et al. [41] developed a dynamic GWP accounting approach considering the temporal profiles of GHG emissions and applied the method to a GWP comparison between corn ethanol and gasoline. The results displayed that considering the carbon temporal effects is critical for qualifying the goal achievement of reducing GWP by using biofuel instead of fossil fuel [41]. Faraca, Tonini and Astrup [44] quantified the GWP of varied wood waste cascading systems with considering the temporal profile of GHG emissions, and concluded that accounting the temporal effects of GHG emissions was critical for biogenic CO_2_ and storage [44]. Yang and Chen [45] applied the dynamic GWP accounting method to syngas production by the gasification system from crop residues in China. The results of the dynamic method showed a discounted GWP mitigation benefit from using crop residues compared to the traditional static method [45]. Daystar et al. [46] applied the GWP accounting approach developed by Levasseur et al. [41] to examine the GWP of ethanol production from six lignocellulosic feedstocks under varied time horizons. The results emphasized the importance of GHG emission temporal profiles and analytical time horizons [46]. However, few of these dynamic GWP studies considered the impacts of variations in forest dynamics and forest management scenarios on the GWP results. Several studies included the carbon dynamics of forest systems in carbon analysis and showed the necessity of including carbon temporal profiles of forest growth [48–55], but these studies used other indicators such as carbon payback time. For example, Sterman, Siegel and Rooney-Varga [48] simulated the replacement of wood for coal in electricity generation and tracked the carbon fluxes in forest, atmosphere, and soil. The payback time for the carbon debt was reported to range from 44 to 104 years, depending on forest type and growth. Following the work by Sterman, Siegeland Rooney-Varga [48], Rolls and Forster [51] replicated the model and modified the input parameters to predict the forest carbon uptake and payback time of the carbon debt with considering the uncertainty in forest growth. This study showed the payback time largely dependent on the uncertainty of forest growth curves [51]. Jonker, Junginger and Faaij [49] calculated the carbon payback period for wood pellets from softwood forest, taking into consideration the temporal profiles of forest growth under several management scenarios. The results highlighted the impacts of forest growth and yield, carbon replacement factor (i.e., how much fossil carbon is avoided by using 1 metric ton biomass carbon), system boundaries, and the baseline selection, on the carbon payback period [49]. Although these studies included the forest carbon dynamics that mainly affect carbon uptake, they did not include the temporal effects of carbon emissions along the biofuel supply chain on the GWP results.

The overall objective of the study is to conduct a dynamic life-cycle carbon analysis allowing for assessing the impacts of forest dynamics and carbon temporal effects on the life-cycle carbon implications of fast pyrolysis biofuels derived from forest residues, which has not been quantified in the literature reviewed above. By mathematically linking key parameters related to the biorefinery operations and alternative forest management scenarios with Monte Carlo simulation and process-based forest growth simulation, the study includes the dynamic profiles of both carbon emissions and uptake. Furthermore, this study developed a time-based discounted GWP method (100-year analysis timeframe) to incorporate the climate effects of carbon emissions/uptake at different years. The results of the dynamic carbon modeling method were compared to the traditional life-cycle carbon analysis relying on the carbon neutrality assumption to examine the impacts of incorporating carbon dynamics. This study aims to provide critical understandings of carbon dynamics across the forest, biorefinery, and biofuel consumption for the design, planning, and optimization of future biomass-to-biofuel systems.

## Results

### Forest growth

**Fig. 1:**
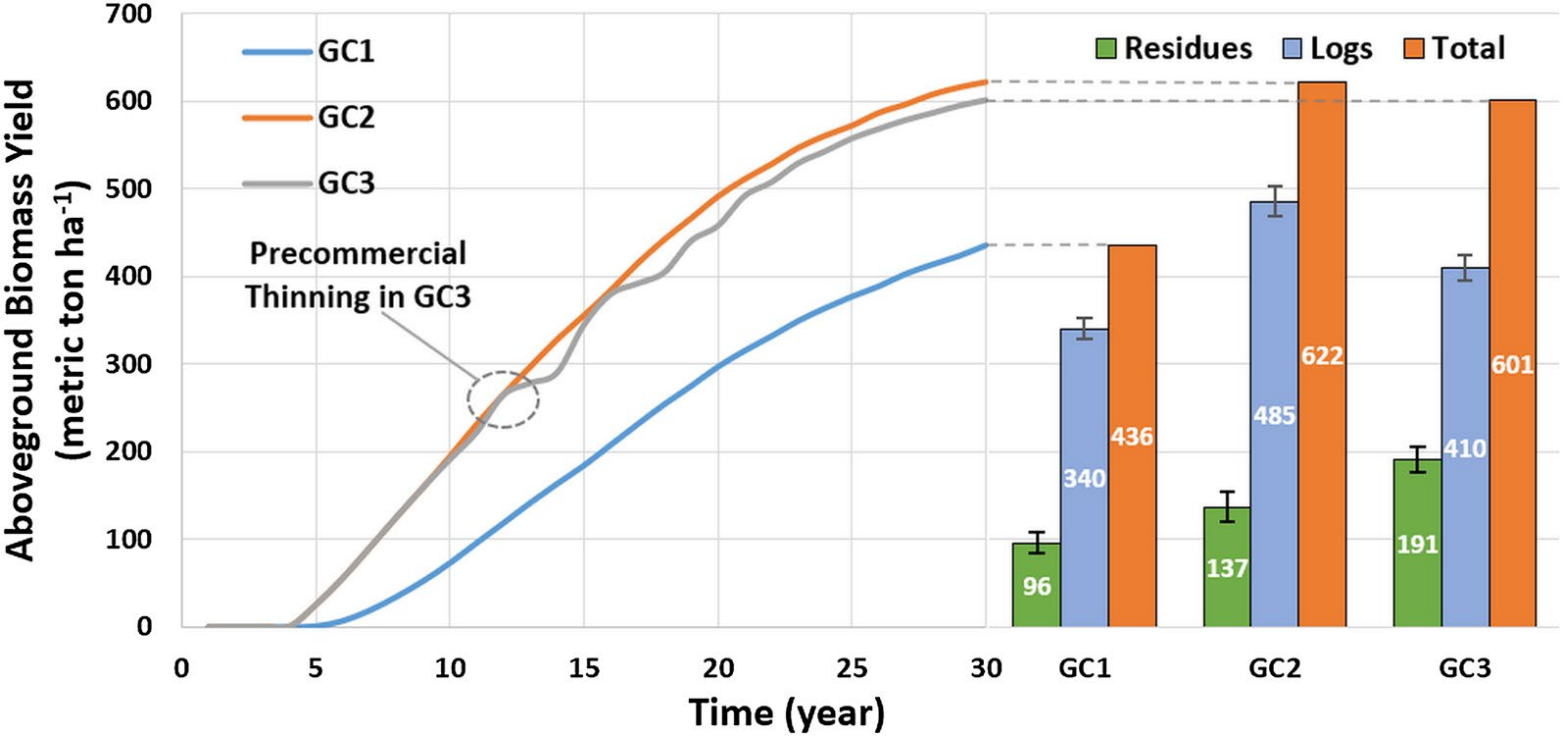
Pine growth of three cases on 1-ha forest land for a rotation of 30 years

This study explored three growth cases (GC1–3) where different GC represents varied site productivity and management strategies (see “Methods” for details). In each case, the aboveground biomass yield was simulated using the FASTLOB simulation and the results are shown in Fig. 1 [56]. The simulation results include the aboveground live tree biomass and total pine residues generated from precommercial thinning per ha forest land for 30 years (1 rotation). The breakdown of logs and residues (mean value) for 1 rotation cycle is plotted in Fig. 1 with error bars showing the 5th–95th percentile (P5–P95) range of the Monte Carlo simulation results. The residues in Fig. 1 include all the pine residues from precommercial thinning and logging, 50%–70% of which are collectible for biofuel production (see Table 1). Among the three cases, GC2 has the highest log output (485 metric ton ha^−1^), while GC3 has the highest residue output (191 metric ton ha^−1^). The reasons are that GC3 has precommercial thinning in year 12 while GC2 has no precommercial thinning and that both GC2 and GC3 have a high site index. GC1 has the lowest outputs for both logs and residues due to the low site index. More temporal details of the LCI data are available in Section “A time-based discounted GWP method for addressing the carbon temporal effect” and Additional file 1: Section "Pine growth and yield".

### Life-cycle GWP on 1-MJ biofuel basis

**Fig. 2:**
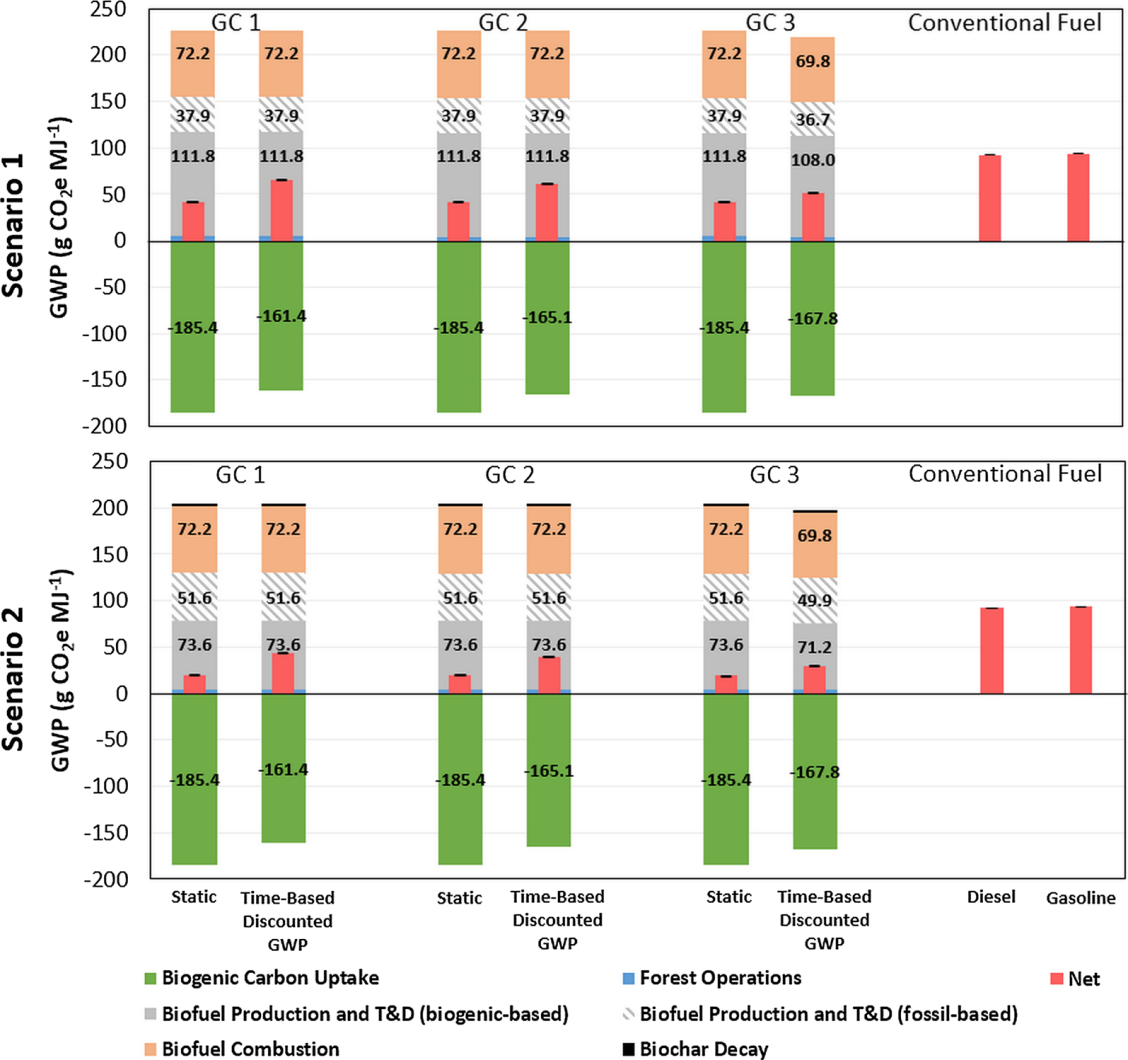
Life-cycle GWP of 1 MJ biofuel using the time-based discounted GWP method in two scenarios compared to conventional fossil fuels (error bar for P5–P95 of the net GWP): Scenario 1 Energy Recovery and Scenario 2 Biochar Utilization. T&D: biomass transportation and fuel distribution

Figure 2 shows the life-cycle GWP of 1-MJ biofuel produced in two scenarios compared to static method cases (see “Scenario analysis”). Scenario 1 combusts the biochar from fast pyrolysis in the CHP for energy recovery, while Scenario 2 explores the potential implications of utilizing the biochar as a soil amendment (see “Scenario analysis” for detailes). In each scenario, three GCs are presented. The error bars represent the P5–P95 range of the Monte Carlo simulation for the net GWP. The time-based discounted GWP method (100-year analysis timeframe) was applied to quantify the carbon temporal effects of life-cycle CO_2_ emissions and sequestration in one rotation cycle. For both scenarios in Fig. 2, the carbon sequestered in the residues via CO_2_ uptake during biomass growth offers significant carbon sequestration credits shown as negative values. Biogenic CO_2_ emissions during biofuel production are a major emission source. Fossil CO_2_ emissions from the combined heat and power (CHP) plant combusting the off-gas from natural gas steam reforming (producing hydrogen) are relatively small compared to the biogenic CO_2_ emissions from the combustion of fuel gas and biochar. Biofuel combustion (use phase) is another major emission source. The GHG emissions associated with forest operations are minor compared to the other components. The annual carbon emissions in two scenarios are documented in Additional file 1: Section "Carbon Emission Profile". GWPs of conventional fuels are obtained from the Greenhouse Gases, Regulated Emissions, and Energy Use in Technologies (GREET) 2019 [57].

According to Scenario 1 in Fig. 2, first, across the three growth cases GC1–3, the differences in the static method are insignificant (< 1.5%) due to two reasons: (1) across GC1–3, the GHG emissions by forest operations on 1-MJ basis are small (4.3–4.7 g CO_2_e MJ^−1^), so the GHG differences brought by different forest management and site productivity are small; (2) the temporal effects of different carbon emission profiles of growth cases are not considered in the static method, so the temporal features of three different growth cases do not differ. Second, using the time-based discounted GWP method increases the GHG emissions given the assumption that the carbon cycle starts with the collection of the pine residues for conversion to and combustion of biofuels prior to the subsequent carbon uptake during biomass growth in a new rotation. Taking Scenario 1 GC3 as an example, the net time-based discounted GHG emissions are 50.2–51.7 MJ^−1^ as P5–P95 compared to the static cases 40.3–41.6 MJ^−1^ as P5–P95. This is mainly due to the differences in biogenic carbon uptake. As the biogenic carbon uptake is sequential and negative in 30 years (see Additional file 1: Fig. S4 and S7), using the time-based discounted GWP method reduces the biogenic carbon uptake and sequestration credits. In GC3, the biogenic carbon uptake in time-based discounted GWP is −167.8 g CO_2_e MJ^−1^ compared to −185.4 g CO_2_e MJ^−1^ in the static method. Third, the different carbon emission profiles in three growth cases also lead to varied GHG results of each component with the time-based discounted GWP method. GC3 biogenic carbon uptake is −167.8 g CO_2_e MJ^−1^ compared to −161.4 g CO_2_e MJ^−1^ in GC1 and −165.1 in GC2. This is determined by the GWP discounting curve with the time-based discounted GWP method (see Additional file 1: Fig. S4) where the value of discounted GWP value decreases in later years of the 100-year analysis timeframe. In GC3, the carbon uptake rate per MJ basis in the first 12 years is much larger than GC1 and GC2 (see Additional file 1: Sect. "Carbon Emission Profile", Fig. S5–S7) due to the precommercial thinning happening in year 12 and felling the trees that are all viewed as residues. Due to the precommercial thinning, 39% (mean value) of the GC3 residue output occurs in year 12 and 61% in year 30 (end of the rotation), compared to 100% of residue output in year 30 in GC1 and GC2. Besides in varying GWP of biogenic carbon uptake, the phenomenon led by different carbon emission profiles is also observed in the varying GWP of biofuel production and biomass transportation and fuel distribution (T&D), and biofuel combustion in different growth cycles. In GC3, year 1 and year 12 have impulse emissions by biofuel production and T&D, and biofuel combustion, while GC1 and GC2 have those emissions in year 1. Hence, time-based discounted GWP method discounts the emissions in year 12 of Scenario 1 GC3 and results in lower GWP results in biofuel production and T&D (144.7 g CO_2_e MJ^−1^), and biofuel combustion (69.8 g CO_2_e MJ^−1^). It is noticeable that, in Scenario 1 GC1 and GC2, GHG emissions of biofuel production and T&D (149.7 g CO_2_e MJ^−1^) and biofuel combustion (72.2 g CO_2_e MJ^−1^) are the same with and without applying the time-based discounted GWP method given the same relative GWP factor, which is 1, in year 1 (starting year). In the comparison of net GWP among the three GCs in Scenario 1, GC3 has the lowest net GWP (51.0 g CO_2_e MJ^−1^) compared to GC1 and 2 (65.2 g CO_2_e MJ^−1^ for GC1 and 61.1 g CO_2_e MJ^−1^ for GC2) with the time-based discounted GWP method. GC3 has the highest yield of forest residues through precommercial thinning that shifts the carbon temporal profile and leads to higher biogenic carbon uptake value and lower carbon emissions compared to GC1 and GC2, as discussed above. This result indicates the need to co-manage forest management and biorefinery production to minimize the life-cycle carbon intensity of biofuels from a life cycle perspective.

In the comparison of Scenario 2 with Scenario 1, the GWP of biogenic carbon uptake, forest operations, and biofuel combustion are the same in the two scenarios since the only difference is the end-of-life of biochar. This difference only impacts the GWP of biofuel production and T&D, and biochar decay (black bars in Fig. 2 Scenario 2). Compared to Scenario 1, Scenario 2 has a 16.4% reduction in the GWP of biofuel production and T&D across all the growth cases and GWP accounting methods due to the carbon stored in biochar instead of being instantaneous release for energy recovery in Scenario 1. This major reduction leads to the decreased net GWP of Scenario 2. For example, in Scenario 2 GC3, the mean net time-based discounted GWP is 29.6 g CO_2_e MJ^−1^ and biofuel production and T&D (including fossil- and biogenic-based GWP) are 121.1 g CO_2_e MJ^−1^ compared to 51.0 g CO_2_e MJ^−1^ in Scenario 1 GC3 that biofuel production and T&D are 144.7 g CO_2_e MJ^−1^. The impact of biochar decay on the net GWP of biofuel is minor as 93.5% of carbon remains in biochar even after 30 years (see Additional file 1: Section "Biochar decay" for details). This result highlights the significant carbon benefits of utilizing biochar from fast pyrolysis biofuel production for soil amendment.

### Life-cycle GWP on 1-ha forest land basis

**Fig. 3:**
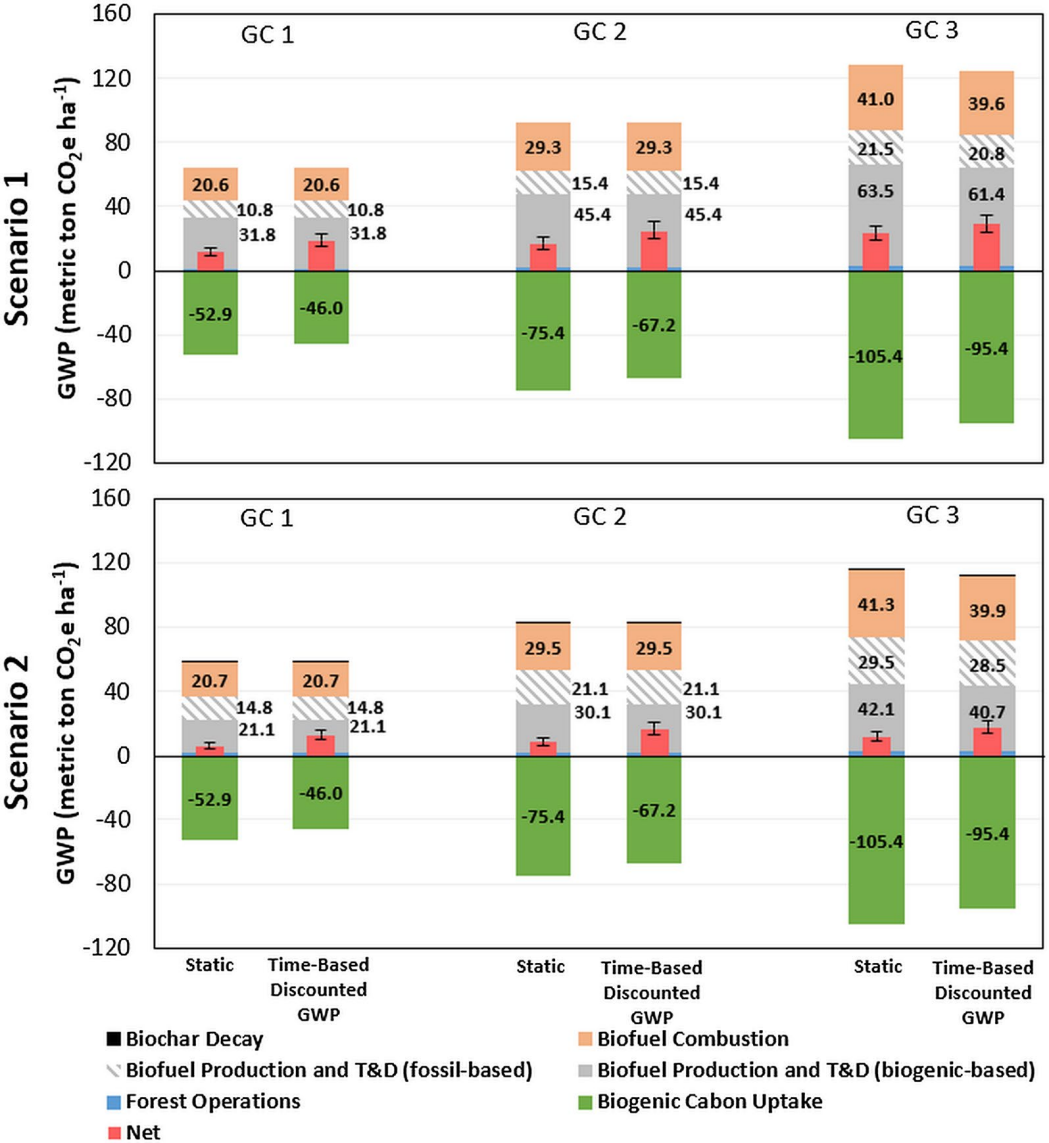
Life-cycle GWP of 1-ha forest land for 1 rotation in two scenarios (error bar for P5–P95 of the net GWP): Scenario 1 Energy Recovery and Scenario 2 Biochar Utilization. T&D: biomass transportation and fuel distribution

This study then conducted extended research on the impacts of varied growth cases on the GWP in two scenarios from the 1-ha perspective by using the residues for biofuel production and combustion in GC1–3 with the time-based discounted GWP compared to the static method (Fig. 3). Although the comparative trends in each growth case of two scenarios are similar to GWP in MJ (Fig. 2), the impacts of forest growth on GWP are more significant on a hectare basis, as demonstrated by the larger differences of GWP across the three growth cases. For example, in Scenario 1, the net GWP of GC3 is 99% higher than that of GC1 in the static method, compared to the negligible difference (< 1.5%) in Fig. 2. Such indications are valuable for conducting landscape-level LCA studies where the balance and optimization of overall carbon stock and fluxes associated with forest growth and harvest/mortality are usually considered. An interesting observation is that GC3 has the highest GWP on 1-ha basis, followed by GC2 and GC1, which contrasts with the results in Fig. 2 where GC1 has the highest GWP on 1-MJ basis. This can be explained by more biofuel outputs in GC3 (due to more forest residues) that lead to more GHG emissions associated with biofuel supply chain.

## Discussion

This work addressed temporal effects associated with key parameters of forest growth, management, and operations in the pine residue-derived biofuel production via fast pyrolysis. Parametric distributions of key life-cycle inventory data encompassing forest growth, management, and operations are developed based on rigorous literature review and process-based engineering modeling and they are incorporated in a dynamic approach to understand the temporal effects of forest dynamics and all carbon emissions and uptake. The results show that forest management has a significant impact on the life-cycle carbon intensity of biofuels when the carbon temporal effects are considered. Such impact is mainly driven by the variations in biomass yields and carbon take of different forest management strategies, while the variations in energy consumption of forest operation have little impact on the biofuel life-cycle carbon intensity. The variations in quality and chemical composition of the pine residues may impact conversion performance, such as biofuel yield and process energy consumption [58–60], which needs further investigation in this field.

This study evaluated a common carbon neutrality hypothesis in LCA of woody-based bioenergy systems by accounting for the carbon temporal effects associated with carbon emissions and sequestration occurring throughout a 30-year pine growth cycle and biofuel production and end use. Using a time-based discounted GWP method, our results show that the time lag of carbon sequestration during pine growth leads to a discounted climate cooling benefit given a timeframe of 100 years from the present. In other words, the time lag results in a somewhat diminishing GHG emission reduction potential of pine residue-derived biofuels compared to a common assumption of carbon neutrality that treats the impacts of carbon sequestration and emissions as static. Importantly, such results are closely tied to our assumption that utilization of readily available pine residues for biofuel production is the starting point of the analysis, given that the bioenergy industry would likely be motivated to start with what is readily available rather than waiting for the completion of a rotation cycle to produce the needed biomass feedstock. This result further highlights the necessity of taking carbon dynamics into account for decision-makers and researchers when investigating the climate change mitigation potential of the bioenergy industry. An overlook of the temporal effects of carbon profiles can weaken the understanding to the role of bioenergy in mitigating global warming, especially for the biomass with long growth.

With the consideration of carbon temporal effects, the GWP results of 1 MJ differ across the three growth cases. These three growth cases present varied carbon profiles in biogenic carbon sequestration and GHG emissions due to the variations in site productivity and management strategies (precommercial thinning or not). However, in the static method cases without considering carbon temporal effects, the mean GWP results of 1 MJ biofuel in each scenario have minor differences (i.e., < 1% for Scenario 1, 2%–4% for Scenario 2). The first reason is that the GHG emissions by forest operations on 1 MJ basis are small. Thus, the GHG differences among different growth cases are small. The second reason is that temporal effects of varied carbon profiles are not considered in the static method. This result emphasizes the importance of considering the forest productivity and management strategies in the future bioenergy LCA research when using dynamic carbon analysis.

Switching the view from 1 MJ basis to 1 ha basis exhibits larger impacts of varied growth cases on the GWP results. The understanding of the carbon impacts coming from variations in site productivity and management strategies can further help future research on varied potential pathways of residues (e.g., pile burning, decay) and forest carbon fluxes in soil and aboveground at a larger scale.

In contrast to combusting the biochar from fast pyrolysis for energy recovery in this study, utilizing the biochar as soil amendment leads to the reduction in the net life-cycle GWP of biofuel. This is mainly caused by the high stability and long-term carbon storage ability of biochar. This carbon storage benefit of biochar outweighs the carbon emissions of electricity purchased to fulfill the energy demand in Scenario 2, while in Scenario 1 such energy demand is met by burning biochar that generates instantaneous carbon emissions. This result can inform future biorefinery design when considering alternative uses of biorefinery byproducts, especially those carbon-beneficial applications beyond energy recovery. In addition to soil amendment, biochar has other applications such as water treatment that can be explored in future research [59].

This study is a stand-level analysis that investigates the carbon flows over a cycle of forest operations occurring on a relatively small defined land area (typically up to hundreds of hectares) that have even-aged trees [49, 50, 61]. In contrast, a landscape-level analysis investigates a much larger scale of the forest that are usually uneven-aged. Thus, forest carbon pools are averaged across trees in different stages of their growth cycles, resulting in a stable carbon level if no change in forest management [49]. The landscape-level analysis may be more appropriate for bioenergy production from managed forest and sustainable forestry management, which aims to maintain an equilibrium of the overall carbon stock and fluxes associated with forest growth and harvest/mortality. A landscape-level LCA may be more appropriate to address managed forests that tend to offer a constant supply of biomass for bioenergy production, among others. The landscape-level analysis will need additional data like the age-distribution of forest and how different forest management strategies change such distributions.

This study also lays the foundation for future research questions with the woody carbon neutrality issue that should be addressed in a deep de-carbonization context. These questions can include the carbon sequestration effects of underground woody biomass production, alternative carbon fates of woody residues that could be left on forest land for decay, and temporal effects of non-CO_2_ climate forcers, such as N_2_O and CH_4_ emissions.

## Conclusions

This study conducted a life-cycle carbon analysis to study the GWP of woody-based bioenergy systems by integrating dynamic carbon modeling with parametric process modeling and Monte Carlo simulation which models the uncertainties associated with the forest growth, management, and operations. The temporal effects of carbon emissions and sequestration from a 30-year pine growth cycle and biofuel production and end use were accounted by adopting the dynamic carbon modeling approach. The study shows that the carbon temporal effect, particularly the time lag of carbon sequestration during pine growth, has direct impacts on the carbon intensity of biofuels produced from pine residues from a stand-level pine growth and management point of view. The significance of such impacts is subject to forest management strategies, end-of-life cases of biochar utilization, and the GHG emission profiles over time. This study also shows the potential carbon benefits of utilizing biochar as soil amendment instead of combusting for energy recovery. The variation in biomass productivities in the three pine growth scenarios would result in a noticeable variation in the carbon intensity of the biofuels when the carbon temporal effect is accounted for. However, it has no impact with the conventional carbon neutrality assumption.

To the best of our knowledge, this is the first dynamic carbon analysis study that addresses carbon temporal effects of pine residue-derived biofuel production at a stand-level. It also considers variations in forest operations and productivity and the impacts on biofuel carbon intensity. This analysis highlights the importance of considering the carbon temporal effects of biofuel carbon intensity in a system involving with woody biomass as a feedstock for biofuel production and may warrant further investigation.

## Methods

### Functional unit and system boundary

**Fig. 4:**
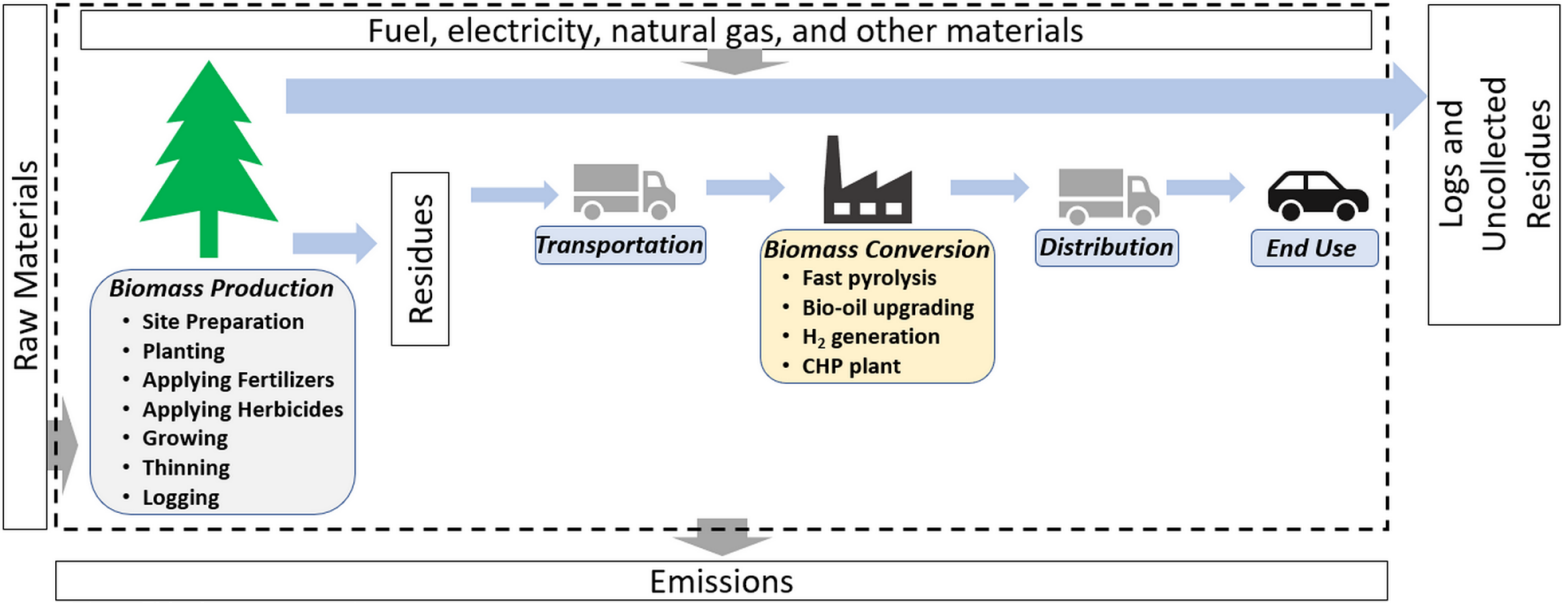
The system boundary of the carbon analysis

In this study, a cradle-to-grave carbon analysis was performed in the Greenhouse Gases, Regulated Emissions, and Energy Use in Technologies (GREET) 2019 model [57] following ISO Standard 14040 series [62] to analyze life-cycle energy consumption and GHG emissions of the biofuel production from pine residues via fast pyrolysis followed by hydroprocessing in the southern U.S. The functional unit is 1 MJ of biofuel produced (to be consistent with traditional LCA studies of biofuels). This study investigates the carbon temporal effects of 1 rotation of pine growth (30 years) in a 100-year analysis timeframe. This study aims to address a key research question, which is, what are the carbon temporal effects of pine residue-derived biofuels 100 years from now? The system boundary for woody biomass-derived biofuels (Fig. 4) included biomass production, biomass transportation, biomass conversion, fuel distribution, and end use (or biofuel combustion). Upstream production of chemicals and fuels used in the biomass production, biomass transportation, biomass conversion, and fuel distribution were also included.

It is assumed that the carbon cycle in this analysis starts with pine residues readily available for conversion to biofuels, followed by carbon sequestration during regeneration of pine that takes 30 years, which is likely the only appealing scenario for starting a new, pine residue-to-biofuel biorefinery. This assumption would lead to an initial carbon debt that would take a long time to pay back, generating delayed carbon sequestration benefits that would only partially compensate the initial carbon debt. On the contrary, if a biorefinery would grow pine first and wait for decades to utilize the woody biomass for biofuel production, the initial carbon sequestration benefit would outweigh the carbon debt that would occur years later.

Energy consumption and emissions from forest management operations, biorefinery operations, and biofuel combustion were included in this analysis. Carbon storage in logs, uncollected residues, and residues for biofuel production was modeled on an annual basis by using a forest growth and yield model under three representative pine management cases (e.g., growth rates and thinning). As this study focuses on the cradle-to-grave system boundary, all the dynamic carbon emissions and sequestration were tracked in detail, and the temporal effects are quantified using a discounted GWP method [63]. As this study focuses on the pine plantation forest which is reforested after each rotation, no land use change is involved in this study. Finally, life-cycle inventory (LCI) data of each life-cycle stage were utilized to run GREET’s Monte Carlo simulation where the pre-defined and mutually independent probability density functions of the key parameters were tested to understand how the uncertainties and variabilities influenced the environmental impacts and carbon analysis results in different scenarios.

### Biomass production

The energy consumption and GHG emissions in the biomass production stage mainly come from forest operations. In this study, fertilized and thinned loblolly pine plantations (FASTLOB), which is a stand-level growth and yield model [56], was used to simulate a loblolly pine (*Pinus taeda L.*) plantation and the associated biomass generated during thinning and final harvesting under different forest operation conditions.

#### Forest operations

Pine growth and yield simulation includes site preparation (including herbicide application), planting, fertilization, thinning, logging, and chipping (more details available in Additional file 1: Section "Forest operations"). While site preparation, planting, and herbicide application are performed at the beginning of one rotation [64], fertilizers are applied twice during the rotation based on the work by Amateis et al. [65]. At the end of a rotation, all the aboveground trees were cut down, generating logs and residues. Thinning is operated as “a silvicultural treatment that reduces tree density primarily to improve tree growth, to enhance forest health, or for economic reasons” [66–68]. As one widely used type of thinning, precommercial thinning commonly takes place at the early stage of a rotation and usually before trees reach merchantable size [69, 70]. Hence, biomass outputs from precommercial thinning are only residues. The benefits of precommercial thinning include preventing stagnation, increasing the stem volume per tree (in other words, produce logs with larger size), and reducing the risk of southern pine beetles [68, 69, 71]. Logging at the end of one rotation and precommercial thinning in the middle of the rotation are the main sources of pine residues [72]. Logging also generates logs that are the main products of forest operations. Balancing the value of the logs obtained and the cost of precommercial thinning is always a complex issue for landowners and depends on many factors such as the availability of sawmills (determining the logistic cost for logs) or wood products manufacturers for logs of different size and quality (determining the selling prices for logs). One study for pine plantation in the southeastern U.S., more specifically Alabama, indicated that forest landowners were encouraged to practice thinning for bioenergy under favorable biomass and/or timber prices [73]. Therefore, this study included the impacts of precommercial thinning into three GCs detailed in a later section (Section “Pine growth and yield”) [74]. Residues collected from thinning and logging were assumed to be chipped on-site and then transported to the biorefinery. Based on the data from the US Department of Agriculture Forest Service for southern softwood, the mass fraction of foliage and branches (residues) were assumed to be 19–25% of the aboveground live tree mass, the rest of which were stem wood and stem bark (logs) [75]. Hence, the residues consist of two portions, all the biomass outputs of precommercial thinning, and 19–25% of the biomass outputs of logging. More details of calculating residue amount are available in Additional file 1: Section "Pine growth and yield". Not all residues were collected to minimize the potential environmental impacts (e.g., biological diversity, risk of soil erosion, feedstock characteristics, machinery technology) [36, 76, 77]. The mass fraction of collectible residues for biofuel production was assumed to be 50–70% with the rest of residues left on forest land [36, 76, 77]. Uncollected residues were not included in the system boundary as shown in Fig. 4. The residues left on the forest land are prone to natural decay which emits GHG [78]. The dynamics of forest residues are complex and could be included in the future analysis as counterfactual scenarios.

The main energy and material requirements of forest operations are diesel fuel for equipment operation and applying the chemicals, e.g., fertilizers and herbicides. The quantity of each input was collected from the literature and statistically analyzed to identify ranges and characterize distributions (Table 1) for Monte Carlo simulation of the impacts of variations in forest operations (see details in Additional file 1: Section "Probability distribution of key parameters"). When only a few data are available, deterministic values were used (Table 2). The energy and GHG (mainly CO_2_, CH_4_, and N_2_O) emissions of upstream production of fuels and chemicals used in forest operations were from the GREET 2019 model [57]. Also, fertilizer-induced N_2_O emissions were accounted for [79]. In this study, mass allocation was used to allocate energy consumption and emissions associated with forest operations between the logs and forest residues (Additional file 1: Section "Forest operations") [61, 80], instead of using system expansion and economic allocation. According to the ISO Standard 14041 “The method of avoiding allocation by expanding the system boundaries is only applicable when the alternative method is known. Assumptions about what is actually replaced by the output of the alternative system shall be well documented. If the conditions cannot be met, the procedure of system expansion is not applicable and allocation will be required” [81]. The southern pine logs in this study do not have an alternative system that producing the logs with the same quality and function. The conditions of using system expansion cannot be met in this study due to the lack of alternative systems to produce logs and forest residues.

**Table 1:** Statistical distribution of parameters with variations in the biomass production models

	Unit	Mean	Minimum ^b^	Maximum ^b^	Distribution
Diesel consumption in site preparation [64, 82–84]	kg ha^−1 a^	72.11	43.65	94.59	Uniform [43.65, 94.59]
Diesel consumption in thinning (with collecting) [64, 82, 84–90]	kg m^−3^ residues	1.02	0.62	1.46	Uniform [0.62, 1.46]
Diesel consumption in logging [64, 82, 83, 85–95]	kg m^−3^ log	1.66	0.37	3.57	Normal *N *(1.40, 0.6^2^)
Collectable pine residue mass fraction [36, 76, 77]	%	60	50	70	Uniform [50, 70]
Mass fraction of residue in the whole tree [75]	%	22	19	25	Uniform [19, 25]

^a^ 1 ha = 10,000 m^2^

^b^ The lower and upper bound values are the lowest and highest data collected from the literature

**Table 2:** Deterministic parameters used in the biomass production models

	Unit	Value
Parameters for forest operations		
Rotation length	Year	30
Diesel consumption of applying fertilizers and herbicides [64]	kg ha^−1 a^	7.5
Diesel consumption of planting [64]	kg ha^−1^	23.4
Diesel consumption of chipping [96]	kg m^−3^ residues	2.8
Diesel consumption of collecting, piling and burning [83]	kg ha^−1^	145.4
Nitrogen fertilizer usage [65]	kg N ha^−1^	103.1
Phosphorus fertilizer usage [65]	kg P_2_O_5_ ha^−1^	12.8
Herbicide usage [64]	kg ha^−1^	1.36
Parameters for biomass outputs		
Aboveground live tree moisture content [97]	% wet	50
Aboveground live tree wet density [98, 99]	kg m^−3^	930
Parameters for biomass outputs		
Pine residue carbon content [57]	%	50.1
Pine residue moisture content [57]	%wet basis	45
Pine residue ash content [57]	%	0.76

^a^ 1 ha = 10,000 m^2^

#### Pine growth and yield

To evaluate the effects of varied site conditions and alternative loblolly pine plantation management practices on stand-level growth and yield, three representative GCs were developed for FASTLOB simulations whiling maximizing the yield of the more valuable pulpwood and sawtimber products (Table 3). The three GCs differ in site productivities (measured by the site index that is a widely accepted indicator representing the site quality and the ability of forest land to grow trees [100, 101]), the timing of the precommercial thinning, and the quantities and schedules of fertilizer application (Additional file 1: Section "Pine growth and yield").

**Table 3:** Growth case (GC) settings

Growth case	Site index	Planting density (trees ha^−1^)	Precommercial thinning	Residual basal area^a^ (m^2^ ha^−1^)	Fertilization application time (after planting)
GC1	60	2152	N/A	N/A	Years 10 and 16
GC2	90	2152	N/A	N/A	Years 10 and 16
GC3	90	2152	Year 12	20.7	Years 13 and 19

^a^Residual basal area is the total area of the tree stem at the base after thinning [102]

The low and high productive sites were represented by typical site indices of 60 and 90, respectively [97]. In all the cases, a high planting density of 2152 trees ha^−1^ was adopted to more closely reflect forest management aimed at maximizing total biomass growth [68, 73]. While the exact thinning prescription (schedule and intensity) depends on various factors, including the goals of the landowner, the location of commercial manufacturing infrastructure, rotation age, forest health and vigor, stands are typically thinned when trees start to compete for light, moisture, and nutrition. Such competition can be measured by stand basal area, among other criteria, and it is considered that a stand is in need of thinning when the basal area exceeds 23.0 to 27.6 m^2^ ha^−1^ [67]. A thinning prescription aimed at maintaining tree growth and vigor and maximizing sawtimber is usually recommended, where pulpwood is thinned to reduce the basal area to a range between 13.8 and 20.7 m^2^ ha^−1^ [67]. Following such recommendation, the thinning intensity was simulated for residual stand basal area of 20.7 m^2^ ha^−1^ at age 12 years. Two applications of fertilization were assumed to occur at ages 10 and 16 when the stand was not thinned (GC1 and GC2). However, for GC3, the fertilization was assumed to occur after thinning. The amount of applied nitrogen and phosphorous fertilizers was based on the literature and documented in Table 2 [65, 73, 103, 104]. Results of the three growth cases are presented in Additional file 1: Table S1, including annual aboveground biomass productivity and removed biomass.

### Biomass transportation

The energy use for the pine residues transported to the biorefinery by trucks was estimated from GREET 2019 [57] where transportation distance 32 miles (51 km), transportation fuel economy at 0.20 gallon mile^−1^ (0.47 l km^−1^), and truck loading capacity of 20.6 metric tons are assumed.

### Biomass conversion

**Fig. 5:**
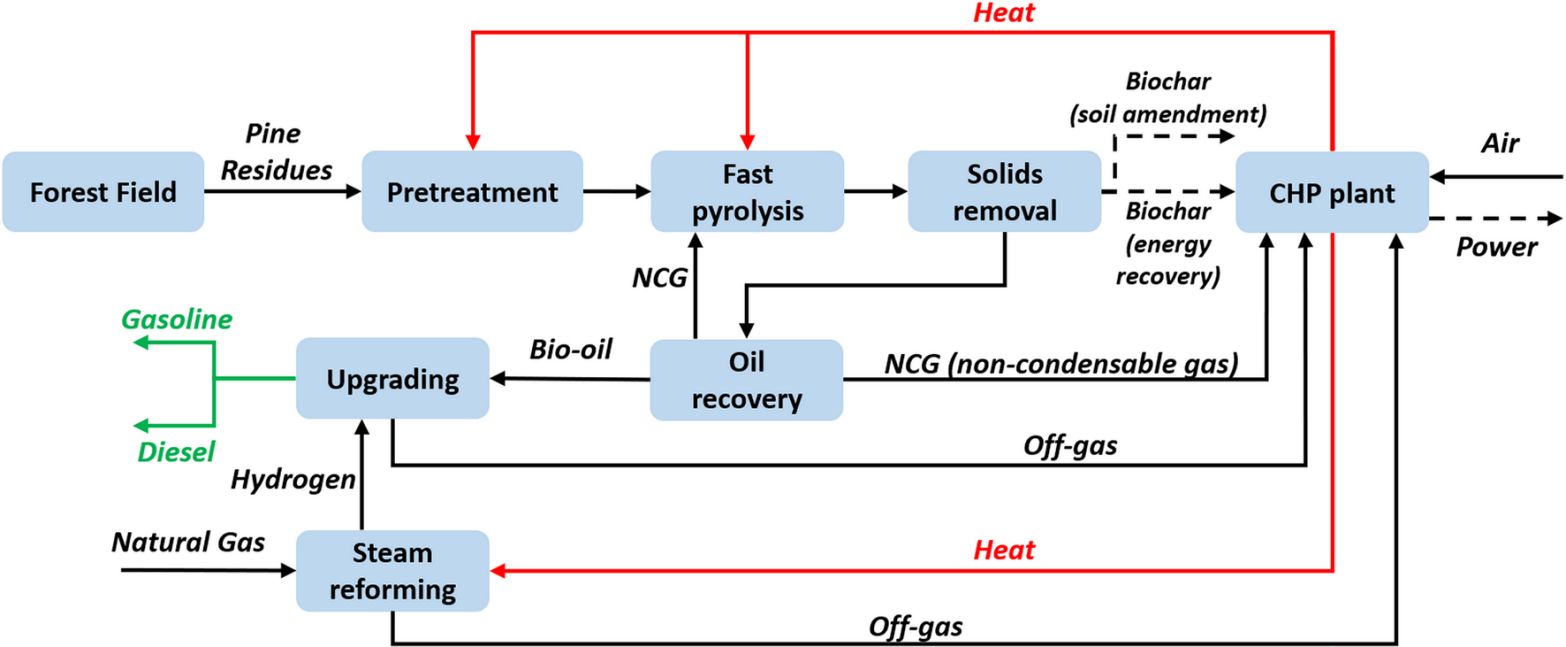
Process flow diagram of fast pyrolysis biorefinery

An Aspen Plus simulation model was developed for the fast pyrolysis biorefinery based on the process model of Ou et al. [105] and operational data collected from the report [32]. The process flow diagram of the model is shown in Fig. 5. The chips from the forest site are first pretreated to reduce the size and moisture content to 9% (*wet basis*) [106]. Size reduction and drying are essential for efficient heat transfer in fast pyrolysis [106, 107]. The heat demand for drying and unit operations was met by the CHP plant inside the biorefinery. The pretreated feedstocks are then sent to fast pyrolysis operated at 500 ℃ and 1 atm pressure in a recirculating fluidized bed reactor. The main products of fast pyrolysis are pyrolytic vapors and solid products such as biochar and sand that are separated by sequential cyclones. Hot sand is sent back to the reactor; biochar with fine particles is sent to CHP for combusting and heat recovery [108]. The pyrolytic vapors from the fast pyrolysis unit are quenched by a two-stage condenser to separate bio-oil from non-condensable gas (NCG). Part of the NCG is sent back to the pyrolyzer as the fluidizing gas, while the rest is sent to the combustor. In this Aspen Plus biorefinery model, the crude bio-oil is upgraded to hydrocarbon fuels by catalytic upgrading [32]. The hydrocarbon outputs are further distilled in two distillation columns to produce gasoline-range and diesel-range liquids as the final fuel products [32]. The hydrogen needed by the process is supplied by the reforming of natural gas (only fossil fuel used in biorefinery in this study) using the steam from the CHP plant. The off-gas from steam reforming is also sent to the CHP plant. Other studies [32, 33] have assumed that the required hydrogen is derived from the biomass vapors, but the costs and complexities of this alternative relative to the common, mature technology of natural gas reforming, make this alternative less attractive.

As biochar can be used for the soil amendment as a potential carbon sink [109, 110], this study also explores an alternative situation where the biochar is used for soil application instead of being combusted in the CHP plant (see Section “Scenario analysis”). In this situation, if the heat demand cannot be met by NCG and off-gas, natural gas is combusted to provide sufficient heat. For the biochar decay, this study adopted the wide accepted exponential model to account the corresponding GHG emissions after biochar application [111]. The decay rate of fast pyrolysis biochar in soil followed the value given by the Intergovernmental Panel on Climate Change (IPCC) [112]. More details are available in Additional file 1: Section "Biochar decay".

Because the CHP plant has multiple fuel inputs (i.e., biochar, process off-gases, and gas products from oil recovery, upgrading, and steam reforming), the carbon emissions must be separately tracked for biogenic carbon emissions from biomass-based fuels and fossil carbon emissions from off-gases from hydrogen production (originated from natural gas). Hence, the total GHG emissions from the CHP plant are tracked based on the carbon mass of input fuels to record the carbon source information. If the CHP plant generated any surplus electricity, it was assumed to be sold to the grid [33]. System expansion was used for this co-product electricity as a method recommended by ISO Standard 14044 to avoid allocation, this method also has been used in similar biorefineries [113–115]. The surplus electricity is assumed to displace U.S. average electricity mix [57].

### Fuel distribution and end use

Diesel and gasoline products are distributed to the market and used in vehicles. The GHG emission factors of fuel distribution and combustion were collected from GREET 2019 [57].

### A time-based discounted GWP method for addressing the carbon temporal effect

A carbon temporal effect analysis was performed to explore the impacts of carbon sequestration and emissions at different time. In order to evaluate the global warming impacts of the delay between carbon emissions and sequestration, this study estimated cumulative global warming effects based on discounted GWP of carbon emissions and sequestration over time.

A delay between the carbon emissions and sequestration from the start of the utilization of pine residues (generated from the previous pine growth cycle for biofuel production) to the completion of a following 30-year rotation cycle of pine growth can vary its global warming effects a given timeframe, e.g., 100 years. Such cumulative global warming effects (*E*_*total*_) are estimated by the sum of the emissions of carbon emissions in a given year *t*, *E*(*t*), multiplied by the discounted global warming potential (dGWP) of the carbon emissions that start in year *t* and continue to exert climate impact until 100 years from now, *dGWP*(100*-t*):

1 $$E_{\text{total}} = \mathop \sum \limits_{t = 0}^{N} E\left( t \right) \times dGWP\left( {100 - t} \right).$$

Here, the term, discounted GWP, is defined relative to the traditional static GWP method in which the GWP of CO_2_ emissions would be considered as 1, regardless of the timing and time horizon of the carbon emissions and carbon sink. *N* is the rotation time (30 years for pine in this study). The dGWP of the emissions in year *t* is the ratio of the absolute global warming potential (AGWP) of the carbon emissions over the subsequent 100*-t* years, *AGWP*_*CO2*_(100*-t*), to that of reference CO_2_ emissions over 100 years from the presence, *AGWP*_*CO2*_(100):

2 $$dGWP_{CO2} \left( t \right) = \frac{{AGWP_{CO2} \left( {100 - t} \right)}}{{AGWP_{CO2} \left( {100} \right)}}.$$

AGWP, in W m^−2^ kg^−1^ year^−1^, is the integration of radiative forcing of a climate forcer in a given time horizon. The AGWP of CO_2_ can be calculated as follows:

3 $$AGWP_{CO2} \left( t \right) = 1.759e^{ - 15} \left[ {a_{0} t + \mathop \sum \limits_{i = 1}^{3} a_{i} \tau_{i} \left( {1 - exp\left( { - \frac{t}{{\tau_{i} }}} \right)} \right) } \right],$$

where 1.759*e*^*−15*^ is the radiative efficiency for CO_2_ in W m^−2^ kg^−1^, *τ*_*i*_ are the perturbation time scales for three modes of redistribution of CO_2_ upon release, *a*_*i*_ are the weighting factors for the effect of each perturbation time scale. *τ*_*i*_ and *a*_*i*_ are estimated by the average values of a set of climate models [116]. $\left[ {a_{0} t + \mathop \sum \limits_{i = 1}^{3} a_{i} \tau_{i} \left( {1 - exp\left( { - \frac{t}{{\tau_{i} }}} \right)} \right)} \right]$ can be recognized as the integral result of the IRF of CO_2_ in the atmosphere [116]. Additional file 1: Fig. S4 shows the dGWP of CO_2_ emissions occurring in year *t* from now and last until 100 years from now as estimated by IPCC. We applied this time-based discounted GWP method to quantify the carbon temporal effect of the carbon emissions and sequestration occurring along the supply chain of the pine residues-derived biofuel production within a 30-year rotation cycle. Note that the temporal effects of emissions from other GHGs such as CH_4_ and N_2_O are not considered given their minimal contributions compared to CO_2_ emissions. For example, the GWP of CH_4_ and N_2_O (after using static GWP-100 characterization factors from IPCC [116]) accounts for only 2% of the total GHG emissions (in CO_2_e) in Scenario 1 static method cases (see “Scenario analysis” for scenario analysis details).

To compare the time-based discounted GWP method with current static LCA practice where temporal effects are not considered, this study included a static method case that used static GWP conversion factors for 100 years from IPCC [116].

To better understand the temporal carbon profile of three GCs, Table 4 summarizes the timeline of the forest operations in three GCs (details available in Additional file 1: Fig. S5–S7). In this study, as mentioned above, the biofuel production of final harvested residues from the previous rotation in each GC is assumed to happen in year 1, or at the end of the previous rotation.

**Table 4:** The timeline of activities within one rotation in three GCs

Year	1	10	12	13	16	19
GC1	Logging and biofuel production (from the previous rotation); Site preparation and planting	Fertilization			Fertilization	
GC2						
GC3			Precommercial thinning and biofuel production	Fertilization		Fertilization

### Scenario analysis

In this study, two scenarios were designed to explore the implications of using biochar or for agriculture application, varied forest growth and yield, and carbon accounting methods. Table 5 summarizes the scenario analysis settings of this study. To account for the variabilities, 1000 iterations were run in the Monte Carlo simulation for each case in each scenario.

**Table 5:** Scenario analysis

Scenarios	Growth cases	Biochar usage	GWP accounting methods
Scenario 1	GC1–3	Combusted in the CHP for energy recovery	Traditional static method; time-based discounted GWP method
Scenario 2		Soil amendment	

## Supplementary Information

**Additional file 1.Figure S1**. The process flow chart of pine residue production. **Figure S2**. The timeline of Growth Case 1 and 2 in one rotation. **Figure S3**. The timeline of Growth Case 3 in one rotation. **Figure S4**. Time-based discounted GWP of CO_2_ for 100 years from the presence. **Figure S5**. Annual CO_2_ emissions and sequestration (mean value) of 1 MJ of biofuel produced in Scenario 1 Growth Case 1. **Figure S6**. Annual CO_2_ emissions and sequestration (mean value) of 1 MJ of biofuel produced in Scenario 1 Growth Case 2. **Figure S7**. Annual CO_2_ emissions and sequestration (mean value) of 1 MJ of biofuel produced in Scenario 1 Growth Case 3. **Figure S8**. Annual CO_2_ emissions and sequestration (mean value) of 1 MJ of biofuel produced in Scenario 2 Growth Case 1. **Figure S9**. Annual CO_2_ emissions and sequestration (mean value) of 1 MJ of biofuel produced in Scenario 2 Growth Case 2. **Figure S10**. Annual CO_2_ emissions and sequestration (mean value) of 1 MJ of biofuel produced in Scenario 2 Growth Case 3. **Table S1**. Accumulative aboveground biomass data for one rotation (metric ton/ha). **Table S2**. Key parameter values and distributions based on literature data.

## Data Availability

All data generated or analyzed during the study are included in this published article, the supplementary information, and open-access GREET model.

## Abbreviations

GHG: Greenhouse gas
LCA: Life Cycle Assessment
DOE: Department of Energy
GWP: Global warming potential
IRF: Impulse response function
GC: Growth case
SI: Supplementary information
CHP: Combined heat and power
GREET: Greenhouse Gases, Regulated Emissions, and Energy Use in Technologies
T&D: Transportation and distribution
LCI: Life-cycle inventory
FASTLOB: Fertilized and thinned loblolly pine plantations
NCG: Non-condensable gas
IPCC: Intergovernmental Panel on Climate Change
dGWP: Discounted global warming potential
AGWP: Absolute global warming potential
