# Supplementary material for: Dynamic life-cycle carbon analysis for fast pyrolysis biofuel produced from pine residues: implications of carbon temporal effects
Source: Biotechnol Biofuels. 2021 Sep 29;14:191. doi: 10.1186/s13068-021-02027-4 (PMC8482607; doi:10.1186/s13068-021-02027-4)
Supplement: Supplementary file 1 — Additional file 1.Figure S1. The process flow chart of pine residue production. Figure S2. The timeline of Growth Case 1 and 2 in one rotation. Figure S3. The timeline of Growth Case 3 in one rotation. Figure S4. Time-based discounted GWP of CO2 for 100 years from the presence. Figure S5. Annual CO2 emissions and sequestration (mean value) of 1 MJ of biofuel produced in Scenario 1 Growth Case 1. Figure S6. Annual CO2 emissions and sequestration (mean value) of 1 MJ of biofuel produced in Scenario 1 Growth Case 2. Figure S7. Annual CO2 emissions and sequestration (mean value) of 1 MJ of biofuel produced in Scenario 1 Growth Case 3. Figure S8. Annual CO2 emissions and sequestration (mean value) of 1 MJ of biofuel produced in Scenario 2 Growth Case 1. Figure S9. Annual CO2 emissions and sequestration (mean value) of 1 MJ of biofuel produced in Scenario 2 Growth Case 2. Figure S10. Annual CO2 emissions and sequestration (mean value) of 1 MJ of biofuel produced in Scenario 2 Growth Case 3. Table S1. Accumulative aboveground biomass data for one rotation (metric ton/ha). Table S2. Key parameter values and distributions based on literature data. [file 13068_2021_2027_MOESM1_ESM.docx]

Supplementary Information

**Dynamic Life-cycle carbon analysis for fast pyrolysis biofuel produced from pine residues: implications of carbon temporal effects**

Kai Lan^1^, Longwen Ou^2^, Sunkyu Park^1^, Stephen S. Kelley^1^, Prakash Nepal^3^, Hoyoung Kwon^2^, Hao Cai^*,2^, Yuan Yao^*,1,4^

*^1^Department of Forest Biomaterials, North Carolina State University, 2820 Faucette Drive, Raleigh, NC, 27606, USA.*

*^2^ Systems Assessment Center, Energy Systems Division, Argonne National Laboratory, 9700 South Cass Avenue, Lemont, IL, 60439, USA. E-mail: hcai@anl.gov*

*^3^ USDA Forest Service, Forest Products Laboratory, 1 Gifford Pinchot Drive, Madison, WI, 53726, USA*

*^4^ Center for Industrial Ecology, Yale School of the Environment, Yale University, 380 Edwards Street, New Haven, CT, 06511, USA*. *E-mail: y.yao@yale.edu*

Page**:** 14

**Table of Contents**

[1. Pine residue production 3](#_Toc62294831)

[1.1 Forest operations 3](#_Toc62294832)

[1.2 Pine growth and yield 4](#_Toc62294833)

[1.3 Probability distribution of key parameters 7](#_Toc62294834)

[2. Time-Based Discounted GWP Method 8](#_Toc62294835)

[3. Carbon Emission Profile 9](#_Toc62294836)

[4. Biochar decay 11](#_Toc62294837)

[Reference 12](#_Toc62294838)

# **1. Pine residue production**

## **1.1 Forest operations**

Fig. S1 displays the system boundary used for pine residue production. All inputs and outputs were based on 1 oven dry metric ton of pine residues. The mass allocation was used to apportion the Life Cycle Inventory (LCI) data between logs and residues.


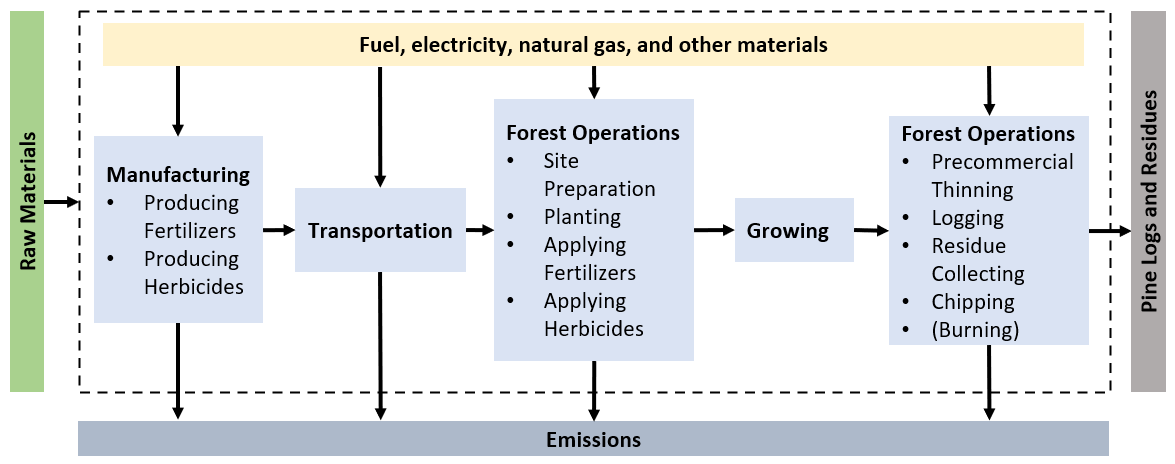


Fig. S1 The process flow chart of pine residue production.

Fig. S2 shows the timeline of the forest operations included in Growth Case 1 and 2; Fig. S3 shows the timeline of Growth Case 3. The different end-of-life cases in each scenario will decide the detailed operations.


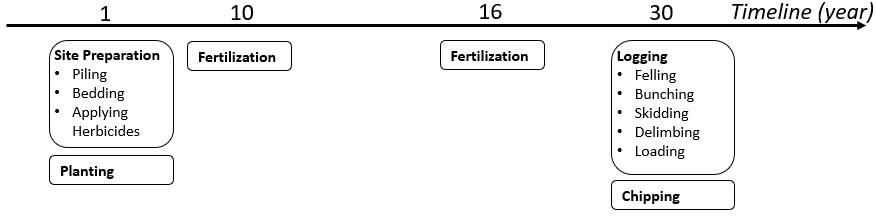


**Fig. S2** The timeline of Growth Case 1 and 2 in one rotation.


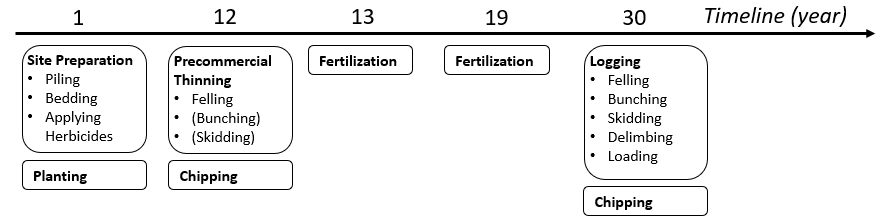


**Fig. S3** The timeline of Growth Case 3 in one rotation.

## **1.2 Pine growth and yield**

The FASTLOB model used in this study has been widely utilized to project stand level characteristics of loblolly pine plantations across the southern U.S., which served as the basis for further analyses to help answer numerous forest management and policy questions [1–5]. The detailed descriptions of data sources and equations and parameters that FATLOB uses in projecting stand-level dominant height, survival, basal area, and biomass volumes are summarized in work by Amateis *et al*. [6].

The number of trees to be planted varies by management objectives (e.g., desired products to be grown), site productivity, and thinning plan, which can vary from as low as 741 trees per ha to up to 2,965 trees per ha [7]. Stand densities are shown to have a substantial effect on the diameter growth of individual trees and as a result, stands with lower planting densities are shown to produce more board foot volumes than stands with higher planting densities [7,8]. A tree planting density of 1,480 to 1,980 trees per ha, depending on site, coupled with thinning are commonly considered as the best compromise where multiple products are the objectives (e.g., pulpwood and sawtimber) [7]. The selected high-end planting density (2,152 trees per ha) represents forest management aimed at maximizing total biomass growth [9].

Table S1 records the accumulative aboveground biomass data and removed biomass amount from thinning in one rotation (30 years) for three scenarios.

**Table S1 Accumulative aboveground biomass data for one rotation (metric ton/ha) ^a^**

|  | Growth Case 1 | | Growth Case 2 | | Growth Case 3 | |
| --- | --- | --- | --- | --- | --- | --- |
| Year | Aboveground biomass | Removed biomass | Aboveground biomass | Removed biomass | Aboveground biomass | Removed biomass |
| 1 | 0 | 0 | 0 | 0 | 0 | 0 |
| 2 | 0 | 0 | 0 | 0 | 0 | 0 |
| 3 | 0 | 0 | 0 | 0 | 0 | 0 |
| 4 | 0 | 0 | 0 | 0 | 0 | 0 |
| 5 | 0.9 | 0 | 25.1 | 0 | 25.1 | 0 |
| 6 | 6.9 | 0 | 55.4 | 0 | 55.4 | 0 |
| 7 | 18.8 | 0 | 89.9 | 0 | 89.9 | 0 |
| 8 | 34.5 | 0 | 124.9 | 0 | 124.9 | 0 |
| 9 | 52.5 | 0 | 158.9 | 0 | 158.9 | 0 |
| 10 | 73.1 | 0 | 194.4 | 0 | 190.8 | 0 |
| 11 | 96.2 | 0 | 231.1 | 0 | 221.9 | 0 |
| 12 | 118.8 | 0 | 265.4 | 0 | 189.0 | 75.5 |
| 13 | 141.9 | 0 | 296.8 | 0 | 202.2 | 75.5 |
| 14 | 163.6 | 0 | 328.0 | 0 | 214.3 | 75.5 |
| 15 | 184.5 | 0 | 355.3 | 0 | 268.8 | 75.5 |
| 16 | 208.0 | 0 | 384.0 | 0 | 303.8 | 75.5 |
| 17 | 231.6 | 0 | 414.7 | 0 | 315.9 | 75.5 |
| 18 | 254.4 | 0 | 442.1 | 0 | 328.6 | 75.5 |
| 19 | 275.3 | 0 | 466.3 | 0 | 364.5 | 75.5 |
| 20 | 297.2 | 0 | 491.2 | 0 | 381.8 | 75.5 |
| 21 | 315.6 | 0 | 511.1 | 0 | 415.8 | 75.5 |
| 22 | 332.2 | 0 | 528.1 | 0 | 431.5 | 75.5 |
| 23 | 349.7 | 0 | 546.8 | 0 | 453.3 | 75.5 |
| 24 | 364.3 | 0 | 560.6 | 0 | 466.7 | 75.5 |
| 25 | 377.5 | 0 | 572.1 | 0 | 481.6 | 75.5 |
| 26 | 389.2 | 0 | 586.7 | 0 | 492.3 | 75.5 |
| 27 | 403.3 | 0 | 596.3 | 0 | 502.4 | 75.5 |
| 28 | 414.0 | 0 | 607.7 | 0 | 510.4 | 75.5 |
| 29 | 423.9 | 0 | 615.8 | 0 | 518.8 | 75.5 |
| 30 | 436.0 | 0 | 621.8 | 0 | 525.3 | 75.5 |

^a^ 1 ha = 10,000 m^2^

The total residues available in each growth case are given by Equation 1 as shown below. All the biomass outputs from precommercial thinning are regarded as residues due to their non-merchantable size at the early stage of the rotation, while 19–25% of the biomass output from logging will be residues (the rest will be logs with bark). For example, in GC3 as shown in Table S1, the residues from precommercial are 75.5 metric tons; the residues from logging are around 525.3 × 22% = 115.6 metric tons. Hence, in GC3, the total residues averagely will be around 190 metric tons.

$Total residues=residues from precommercial thinning+residues from logging$ (1)

## **1.3 Probability distribution of key parameters**

As shown in Table 1 in the main context Section 5.2.1, there are 5 parameters and their distributions that were studied. The chi-square goodness-of-fit tests were conducted in Matlab at 5% significance level to identify the probability distribution of data samples [10]. Equation 2 described the probability density function for normal distribution [11,12]. If the parameter value data points were too few or did not follow the two distributions, then uniform distribution was assumed in Table S2. These assumed distributions can be modified by researchers when more data are available in the future.

$f_{normal}=\frac{1}{\sqrt{2\pi}\sigma}e^{-\frac{1}{2}{(\frac{x-\mu}{\sigma})}^{2}}$ (2)

**Table S2 Key parameter values and distributions based on literature data**

|  | Unit | Test Results | Assigned Distribution |
| --- | --- | --- | --- |
| Diesel consumption in site preparation[13–16] | kg ha^-1 a^ | N/A | Uniform  [43.65, 94.59] |
| Diesel consumption in thinning (with collecting) [13,14,16–22] | kg m^-3^ residue | Fail | Uniform  [0.62, 1.46] |
| Diesel consumption in logging[13–15,17–27] | kg m^-3^ log | Normal | Normal  *N*(1.40, 0.6^2^) |
| Collectable pine residue mass fraction [28–30] | % | N/A | Uniform  [50,70] |
| Mass fraction of residue in the whole tree[31] | % | N/A | Uniform  [19,25] |

^a^ 1 ha = 10,000 m^2^

## **2. Time-Based Discounted GWP Method**

Fig. S4 shows the GWP factors of CO_2_ for 100 year using time-based discounted GWP method.

**
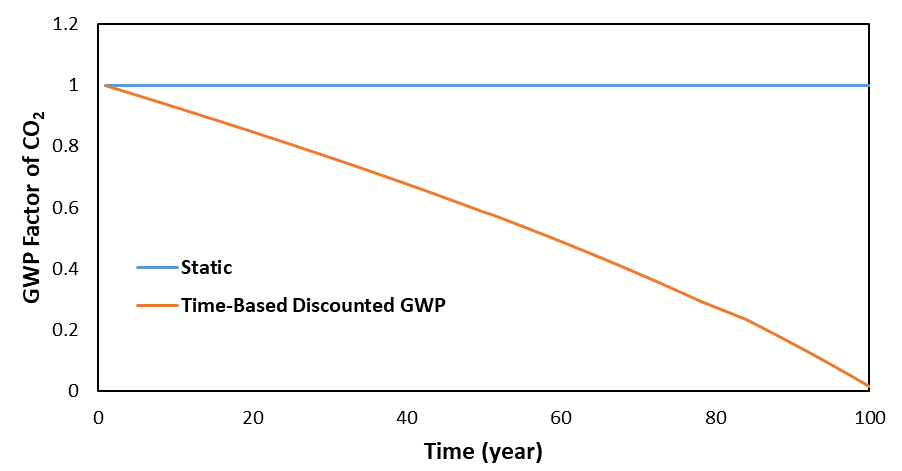
**

**Fig. S4** Time-based discounted GWP of CO_2_ for 100 years from the presence.

# **3. Carbon Emission Profile**

**
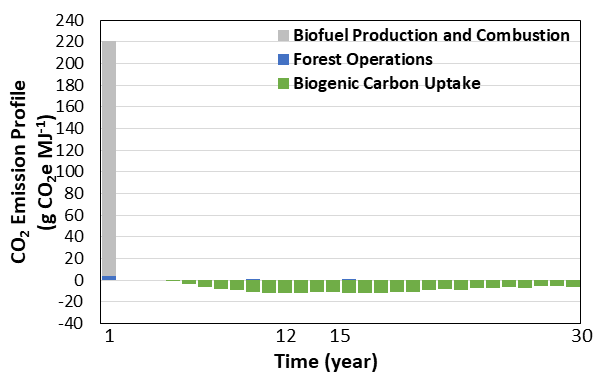
**

**Fig. S5** Annual CO_2_ emissions and sequestration (mean value) of 1 MJ of biofuel produced in Scenario 1 Growth Case 1.


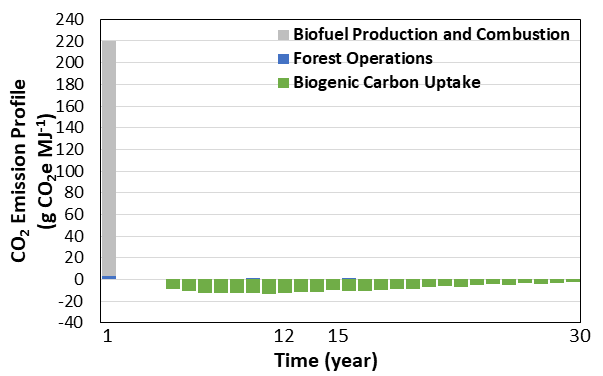


**Fig. S6** Annual CO_2_ emissions and sequestration (mean value) of 1 MJ of biofuel produced in Scenario 1 Growth Case 2.


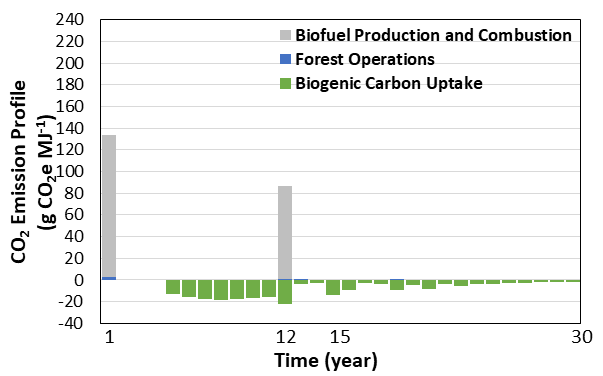


**Fig. S7** Annual CO_2_ emissions and sequestration (mean value) of 1 MJ of biofuel produced in Scenario 1 Growth Case 3.

**
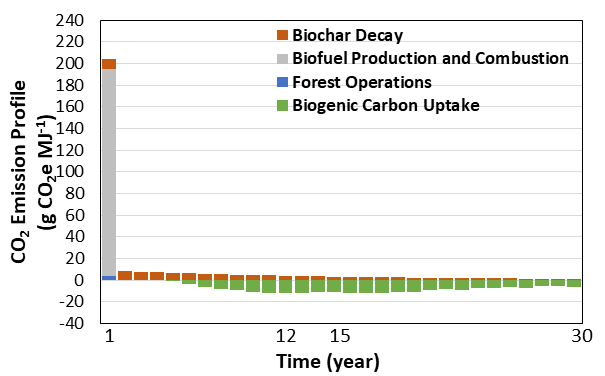
**

**Fig. S8** Annual CO_2_ emissions and sequestration (mean value) of 1 MJ of biofuel produced in Scenario 2 Growth Case 1.


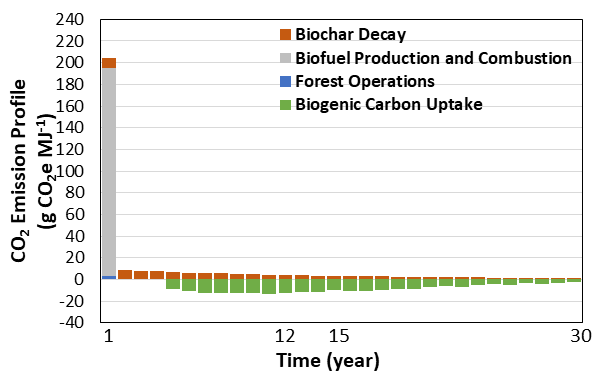


**Fig. S9** Annual CO_2_ emissions and sequestration (mean value) of 1 MJ of biofuel produced in Scenario 2 Growth Case 2.


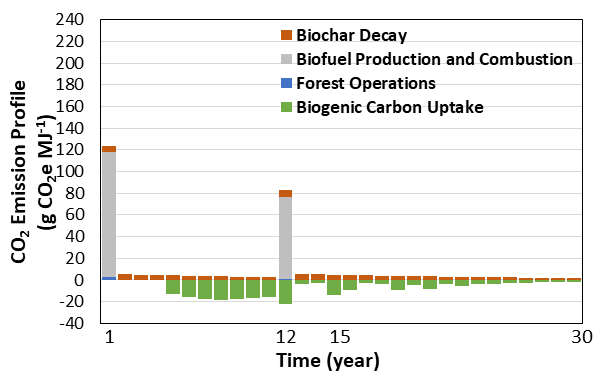


**Fig. S10** Annual CO_2_ emissions and sequestration (mean value) of 1 MJ of biofuel produced in Scenario 2 Growth Case 3.

# **4. Biochar decay**

This study adopts the wide accepted exponential decay model for the biochar after soil application [32]. Equation 3 depicts the remaining carbon mass of biochar after *t* years.

$C_{t}=C_{0}e^{-kt}$ (3)

*C_t_* is the remaining carbon mass of biochar at year *t* after applying biochar to the soil; *C_0_* is the initial carbon mass of biochar at year 0; *t* is decay rate and estimated by the mean residence time of biochar [32]. In this study, *C_0_* was derived from the ASPEN Plus simulation results. For the decay rate *k*, this study follows the practice given by the Intergovernmental Panel on Climate Change (IPCC) [33]. The remaining fraction of medium temperature pyrolysis biochar after 100 years is 80% for cropland or grassland applications. Hence, *k* used in this study is 0.00223.

# **Reference**

1. Stoots BG, Straka TJ, Phillips SL. State-level forestry cost-share programs and economic impact of increased timber outputs: a South Carolina case study. Resources. 2017;6.

2. Christopher SF, Schoenholtz SH, Nettles JE. Water quantity implications of regional-scale switchgrass production in the southeastern U.S. Biomass and Bioenergy. 2015;83:50–9.

3. Shrestha P, Stainback GA, Dwivedi P. Economic impact of net carbon payments and bioenergy production in fertilized and non-fertilized loblolly pine plantations. Forests. 2015;6:3045–59.

4. Wang HJ, Radtke PJ, Prisley SP. Long-term simulations of forest management impacts on carbon storage from loblolly pine plantations in the Southern U.S. USDA Forest Service, Southern Research Station, Asheville, NC, USA; 2012. Available from: https://www.fs.usda.gov/treesearch/pubs/41015. Accessed 11 Apr 2020.

5. Kevin McIntyre R, Jack SB, Mitchell RJ, McCall BB. Financial feasibility of selection-based multiple-value management on private lands in the south: A heuristic case study approach. Journal of Forestry. 2010;108:230–7.

6. Amateis RL, Burkhart HE, Lee Allen H, Montes C. FASTLOB (A Stand-Level Growth and Yield Model for Fertilized and Thinned Loblolly Pine Plantations). 2001. Available from: https://fmrc.frec.vt.edu/content/dam/fmrc_frec_vt_edu/documents/fastlob.pdf. Accessed 11 May 2020.

7. Nebeker TE, Hodges JD, Karr BK, Moehring DM. Thinning practices in southern pines-with pest management recommendations. USDA Forest Service. 1985. Available from: http://ageconsearch.umn.edu/bitstream/156818/2/tb1703.pdf. Accessed 19 May 2019.

8. Dickens ED, Moorhead DJ. A guide to thinning pine plantations. University of Georgia. 2015. Available from: https://bugwoodcloud.org/bugwood/productivity/pdfs/Guide_thinning_pine_plantations.pdf. Accessed 19 May 2020.

9. Gallagher T V., Kantavichai R, Teeter LD. An Economic Analysis of Incorporating Biomass Thinning into Loblolly Pine Plantations in Alabama. Open Journal of Forestry. 2017;07:172–87.

10. Hosmer D.W. and Lemeshow S. Goodness of fit tests for the multiple logistic regression model: Communications in Statistics. Communications in Statistics - Theory and Methods. 1980;9:1043-1069.

11. Stacy EW. A generalization of the gamma distribution. The Annals of mathematical statistics. 1962;33:1187–92.

12. Rosenkrantz WA. Introduction to probability and statistics for scientists and engineers. New York: McGraw-Hill; 1997.

13. Markewitz D. Fossil fuel carbon emissions from silviculture: Impacts on net carbon sequestration in forests. Forest Ecology and Management. 2006;236:153–61.

14. Dwivedi P, Alavalapati JR, Susaeta A, Stainback A. Impact of carbon value on the profitability of slash pine plantations in the southern United States: an integrated life cycle and Faustmann analysis. Canadian Journal of Forest Research. 2009;39:990–1000.

15. Oneil EE, Johnson LR, Lippke BR, McCarter JB, McDill ME, Roth PA, et al. Life-Cycle Impacts of Inland Northwest and Northeast/North Central Forest Resources. Wood and Fiber Science. 2010;42:29–51.

16. Kilpeläinen A, Alam A, Strandman H, Kellomäki S. Life cycle assessment tool for estimating net CO2exchange of forest production. GCB Bioenergy. 2011;3:461–71.

17. Johnson L, Lippke B, Oneil E. Modeling Biomass Collection and Woods Processing Life-Cycle Analysis. Forest Product Journal. 2012;62:258–272.

18. Karjalainen T. Greenhouse gas emissions from the use of primary energy in forest operations and long-distance transportation of timber in Finland. Forestry. 1996;69:215–28.

19. Lindholm E. Energy Use and Environmental Impact of Roundwood and Forest Fuel Production in Sweden. Energy. 2010.

20. Alam A, Kilpeläinen A, Kellomäki S. Impacts of initial stand density and thinning regimes on energy wood production and management-related CO_2_ emissions in boreal ecosystems. European Journal of Forest Research. 2012;131:655–67.

21. Whittaker C, Mortimer N, Murphy R, Matthews R. Energy and greenhouse gas balance of the use of forest residues for bioenergy production in the UK. Biomass and Bioenergy. Elsevier Ltd; 2011;35:4581–94.

22. González-García S, Berg S, Feijoo G, Moreira MT. Environmental impacts of forest production and supply of pulpwood: Spanish and Swedish case studies. International Journal of Life Cycle Assessment. 2009;14:340–53.

23. Karjalainen T, Kellomäki S, Pussinen A. Role of wood-based products in absorbing atmospheric carbon. Silva Fennica. 1994;28:67-80.

24. Klvac R, Skoupy A. Characteristic fuel consumption and exhaust emissions in fully mechanized logging operations. Journal of Forest Research. 2009;14:328–34.

25. Athanassiadis D, Lidestav G, Wästerlund I. Fuel, Hydraulic Oil and Lubricant Consumption in Swedish Mechanized Harvesting Operations, 1996. Journal of Forest Engineering. 1999;10:59–66.

26. Saud P, Wang J, Lin W, Sharma BD, Hartley DS. A life cycle analysis of forest carbon balance and carbon emissions of timber harvesting in West Virginia. Wood and Fiber Science. 2013;45:250–67.

27. Mcnamee P, Adams PWR, McManus MC, Dooley B, Darvell LI, Williams A, et al. An assessment of the torrefaction of North American pine and life cycle greenhouse gas emissions. Energy Conversion and Management. 2016;113:177–88

28. Riffell S, Verschuyl J, Miller D, Wigley TB. Biofuel harvests, coarse woody debris, and biodiversity - a meta-analysis. Forest Ecol Manag. 2011;261:878–87.

29. Langholtz MH, Stokes BJ, Eaton LM. 2016 Billion-Ton Report: Advancing Domestic Resources for a Thriving Bioeconomy, Volume 1: Economic Availability of Feedstocks. Oak Ridge National Laboratory, Oak Ridge, TN, USA; 2016. Available from: https://www.energy.gov/sites/prod/files/2016/12/f34/2016_billion_ton_report_12.2.16.pdf. Accessed 18 May 2020.

30. Fritts SR, Moorman CE, Hazel DW, Jackson BD. Biomass Harvesting Guidelines affect downed woody debris retention. Biomass and Bioenergy. 2014;70:382–91

31. Jenkins JC, Chojnacky DC, Heath LS, Birdsey RA. National-Scale Biomass Estimators for United States Tree Species. Forest Sci. 2003;49:12–35.

32. Lehmann J, Joseph S, editors. Biochar for environmental management: science, technology and implementation. New York, NY: Routledge; 2015.

33. IPCC. 2019 Refinement to the 2006 IPCC Guidelines for National Greenhouse Gas Inventories. Buendia EC, Tanabe K, Kranjc A, Baasansuren J, Fukuda M, Ngarize S, et al., editors. Switzerland: IPCC; 2019. Available from: https://www.ipcc.ch/site/assets/uploads/2019/12/19R_V0_01_Overview.pdf. Accessed 18 Jan 2021.
